# Genomic legacy of migration in endangered caribou

**DOI:** 10.1371/journal.pgen.1009974

**Published:** 2022-02-10

**Authors:** Maria Cavedon, Bridgett vonHoldt, Mark Hebblewhite, Troy Hegel, Elizabeth Heppenheimer, Dave Hervieux, Stefano Mariani, Helen Schwantje, Robin Steenweg, Jessica Theoret, Megan Watters, Marco Musiani

**Affiliations:** 1 Faculty of Environmental Design, University of Calgary, Calgary, Alberta, Canada; 2 Department of Ecology & Evolutionary Biology, Princeton University, Princeton, New Jersey, United States of America; 3 Wildlife Biology Program, Department of Ecosystem and Conservation Sciences, College of Forestry and Conservation, University of Montana, Missoula, Montana, United States of America; 4 Yukon Department of Environment, Whitehorse, Yukon, Canada; 5 Fish and Wildlife Stewardship Branch, Alberta Environment and Parks, Grande Prairie, Alberta, Canada; 6 School of Natural Sciences and Psychology, Liverpool John Moores University, Liverpool, United Kingdom; 7 Wildlife and Habitat Branch, Ministry of Forests, Lands, Natural Resource Operations and Rural Development, Government of British Columbia, Nanaimo, British Columbia, Canada; 8 Pacific Region, Canadian Wildlife Service, Environment and Climate Change Canada, Delta, British Columbia, Canada; 9 Land and Resource Specialist, Fort St. John, British Columbia, Canada; 10 Department of Biological Sciences, Faculty of Science and Veterinary Medicine (Joint Appointment), University of Calgary, Calgary, Alberta, Canada; Case Western Reserve University School of Medicine, UNITED STATES

## Abstract

Wide-ranging animals, including migratory species, are significantly threatened by the effects of habitat fragmentation and habitat loss. In the case of terrestrial mammals, this results in nearly a quarter of species being at risk of extinction. Caribou are one such example of a wide-ranging, migratory, terrestrial, and endangered mammal. In populations of caribou, the proportion of individuals considered as “migrants” can vary dramatically. There is therefore a possibility that, under the condition that migratory behavior is genetically determined, those individuals or populations that are migratory will be further impacted by humans, and this impact could result in the permanent loss of the migratory trait in some populations. However, genetic determination of migration has not previously been studied in an endangered terrestrial mammal. We examined migratory behavior of 139 GPS-collared endangered caribou in western North America and carried out genomic scans for the same individuals. Here we determine a genetic subdivision of caribou into a Northern and a Southern genetic cluster. We also detect >50 SNPs associated with migratory behavior, which are in genes with hypothesized roles in determining migration in other organisms. Furthermore, we determine that propensity to migrate depends upon the proportion of ancestry in individual caribou, and thus on the evolutionary history of its migratory and sedentary subspecies. If, as we report, migratory behavior is influenced by genes, caribou could be further impacted by the loss of the migratory trait in some isolated populations already at low numbers. Our results indicating an ancestral genetic component also suggest that the migratory trait and their associated genetic mutations could not be easily re-established when lost in a population.

## Author summary

Genetic determination of migration has not previously been studied in an endangered terrestrial mammal. The use of global positioning system (GPS) transmitters permitted the detection of migratory or sedentary movements of 139 endangered caribou. The presence of both migratory and resident phenotypes in sympatry allowed for us to examine genomic differences among individuals that had experienced similar environments prior to the initiation of migration. In this study, we genotyped 139 caribou using RAD sequencing, and used these genotypes to assess population structure and investigate potential genetic associations with migration. We detected >50 SNPs associated to migration. These SNPs were found in genes with hypothesized roles in determining migration in other organisms. In addition, propensity to migrate depended upon the proportion of Northern or Southern ancestry in individual caribou, and thus on the evolutionary history of its migratory and sedentary subspecies dating back to the last glaciation. We believe that our concerns for the loss of migration in caribou are transferable to other species and systems where there are documented declines, and migration is likewise associated with genes. If–as we reported–migratory behavior is determined by ancestral genes, species could be further impacted, possibly by the loss of the migratory trait in some populations already at low numbers. This loss could perhaps be averted with the maintenance of critical seasonal habitats within and between seasonal ranges.

## Introduction

Migration, the directional movement from one location to another and back, is observed in numerous species of vertebrates [1], including approximately 36% of marine mammals and about 1% of terrestrial mammals [2]. Migration allows animals to exploit seasonally and geographically variable resources (e.g. food, habitat, favorable climate, or breeding conditions) or avoid unfavorable conditions (e.g. predators, disease) [3–5]. Wide-ranging animals, including several migratory species, are severely threatened by the effects of habitat fragmentation (for example, through the barriers encountered on migratory routes) and habitat loss [6,7].

The bases of animal migration, and the bases of its suite of characteristics (e.g. tendency to migrate or not, timing, direction, and distance), remain largely unknown. Speculation on an innate genetic program [8,9] has been supported by species with known genetic traits that prompt the initiation of migration or minimize the cost of locomotion for efficient migration [10–12]. Migratory behavior could also be learned or dependent on physiological and nutritional conditions [13–15], or even triggered by environmental ones [12,16]. Moreover, migratory behavior could be influenced by the interaction of several of the aforementioned components [12,16,17].

Detecting and quantifying the phenotype and patterns of migration can be immensely challenging, with variation known to occur in the timing, direction, and distance of migration [3]. Application of GPS transmitters [18] to migratory species may reveal additional complexities. For example, some species may be partially migratory, where within the same population only a fraction of individuals migrate [19,20]. Despite the added complexity, the presence of both migratory and resident phenotypes in the same population also allows for the testing of genetic differences among individuals, while assuming exposure to an identical seasonal environment.

Several ungulates are partially migratory [21,22], and there is currently a lack of information regarding the potentially fundamental genetic basis producing migratory and resident individuals within these species’ partially migratory populations [22]. Genomic studies in partial migratory populations have been conducted primarily in fish, birds, and insects [12,23], whereas in ungulates, studies have been mainly based on a handful of neutral molecular markers [24,25]. Partial migration is present in all subspecies and ecotypes (see [26] for a definition) of caribou (*Rangifer tarandus*), but in different proportions. For example, caribou living in forested areas are thought to be mainly sedentary, whereas caribou living in the tundra are considered primarily migratory [27]. Migration allows caribou to access seasonally and geographically variable resources, as well as avoid unfavorable conditions [28]. For example, during migration, Barren-ground caribou may move in large aggregations, and it is understood that this herding behavior results in dilution of predation risk per unit caribou [27].

The two behaviors (propensity to migrate and propensity for being sedentary) likely emerged during glacial eras when separate caribou lineages evolved north (Beringian–Eurasian lineage—BEL) and south (North America lineage—NAL) of the continental ice sheet, in areas dominated by tundra and forests, respectively [24,29]. However, following the last deglaciation, a post-secondary contact between the two lineages occurred, resulting in hybrid zones, such as in the Rocky Mountains. There, the probability of being migratory is higher in individuals carrying mitochondrial haplotypes of the BEL type [24], suggesting a possible genetic determination of migration, but not ruling out a cultural component, as calves grow up with their mothers and mtDNA is maternally inherited.

Caribou are wide-ranging and migratory, and many of their populations (also referred to as “herds”, as they might not be genetically or ecologically distinct) are threatened, endangered, or already extirpated [26,30]. In threatened populations of caribou, the proportion of individuals classified as “migrants” can vary dramatically, with some populations reported as being either fully migratory or fully sedentary [24], and Williams et al. [31] claimed that habitat loss is responsible of the endangerment of migratory caribou in particular. There is therefore a possibility that, under the condition that migratory behavior is genetically determined, those individuals or populations that are migratory will be further impacted by humans (compared to species were migration is not genetically determined). This impact could result in the permanent loss of the migratory trait in some populations. However, despite this concern, genetic determination of migration has not previously been studied in caribou, or in other threatened terrestrial mammals.

**Fig 1:**
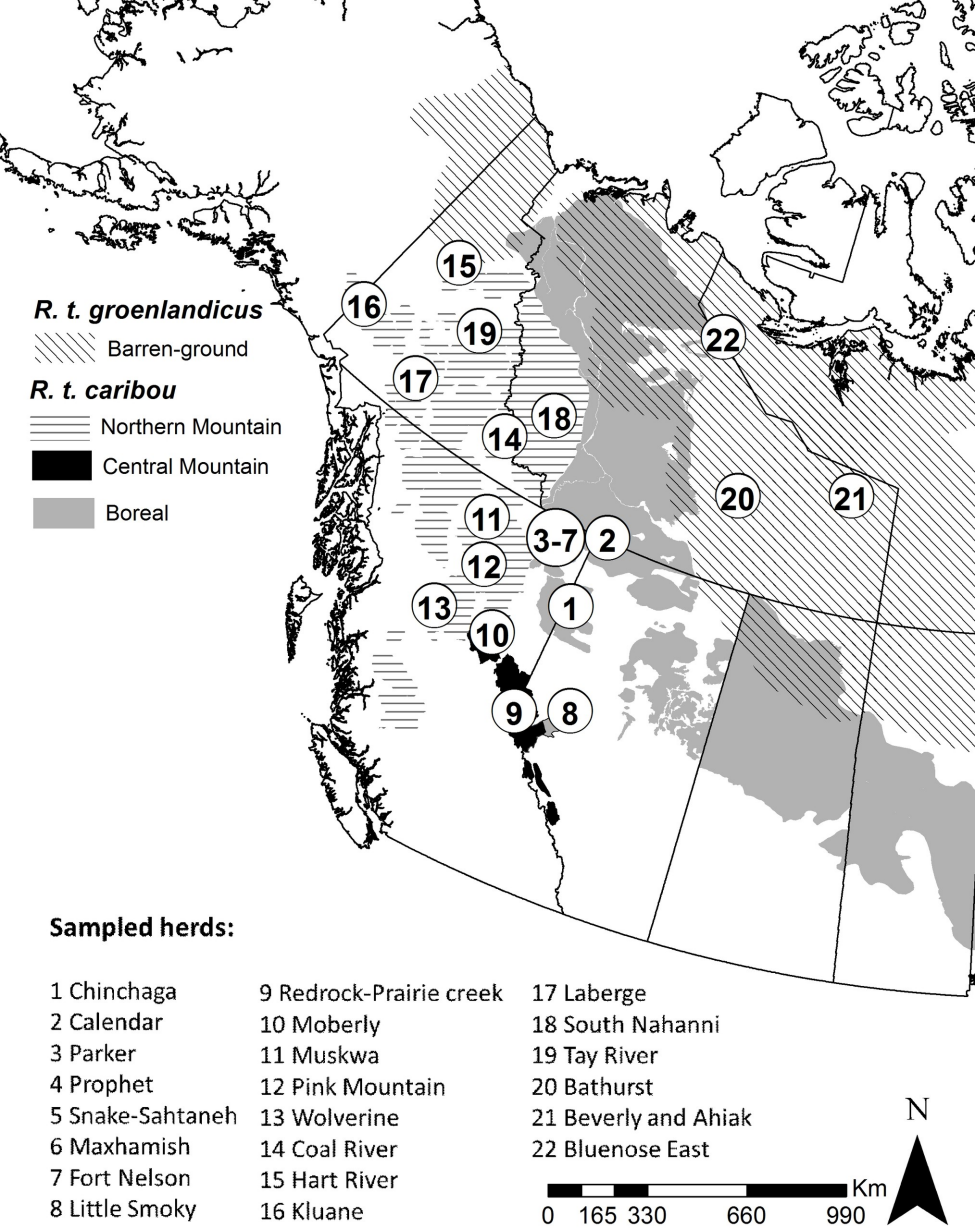
Caribou sampled in western North America. Black numbered circles indicate sampled populations (also referred to as “herds”, as they might not be genetically or ecologically distinct). Grey-scale polygons show the distribution of subspecies and ecotypes: diagonal black lines represent the Barren-ground subspecies (*R*. *t*. *groenlandicus*); horizontal lines, light gray, and black represent Northern Mountain, Boreal and Central Mountain, ecotypes, respectively, within the Woodland caribou subspecies (*R*. *t*. *caribou*). Basemap layers available from: https://www12.statcan.gc.ca/census-recensement/2011/geo/bound-limit/bound-limit-eng.cfm and https://international.ipums.org/international/gis.shtml.

We studied the genetic basis of migration in endangered caribou. We examined western North American caribou belonging to three ecotypes (Boreal, Central Mountain, and Northern Mountain) within the Woodland subspecies (*R*. *t*. *caribou*). We also examined caribou belonging to the Barren-ground ecotype, which forms its own subspecies (*R*. *t*. *groenlandicus–*Fig 1). Here, we aimed at determining seasonal movement and genomic variation of single nucleotide polymorphisms (SNPs) in 139 caribou individuals (Fig 1 and Fig A in S1 Text). We also aimed at detecting associations between migratory behavior and genes known to influence migratory tendency in other organisms. Finally, we aimed at testing whether the propensity to migrate also depended upon the proportion of Northern or Southern ancestry in individual caribou, and thus on the evolutionary history of its migratory and sedentary subspecies.

## Results

### Genetic structure: Two main caribou clusters detected

**Fig 2:**
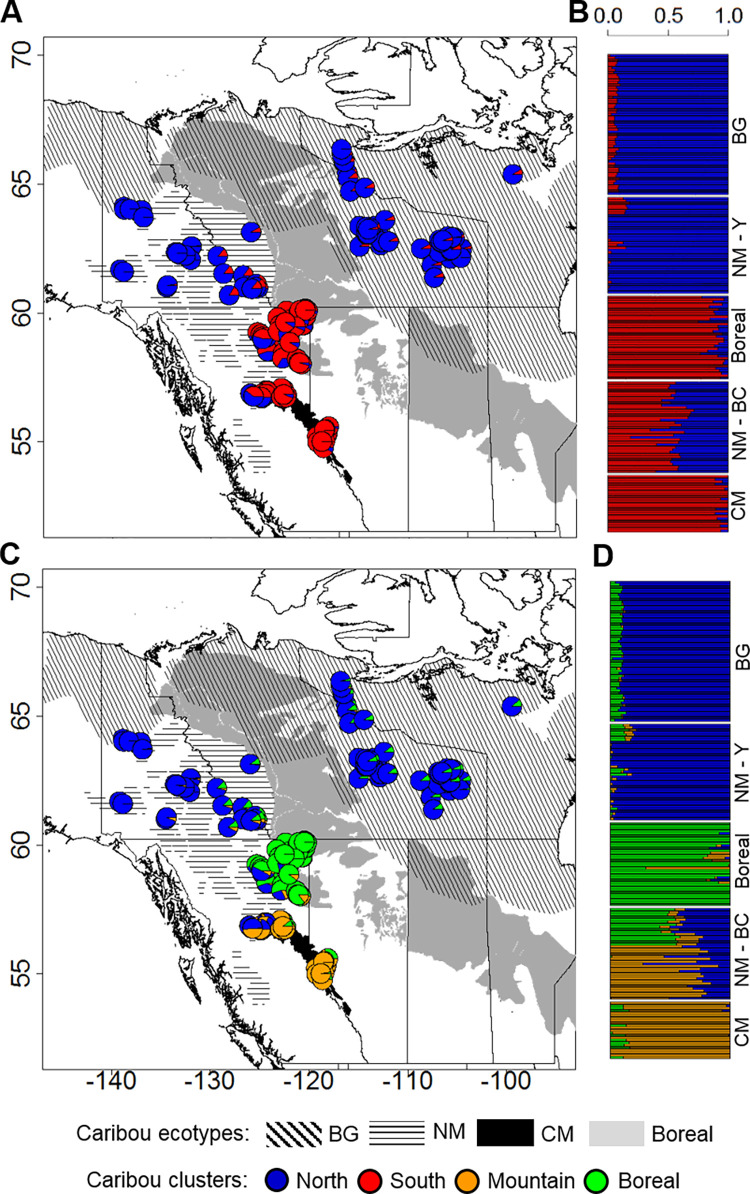
Caribou genetic clusters in western North America. (A)/(B) and (C)/(D) show the best and second best number of clusters (K = 2 or 3, respectively) describing population structure. Further subdivisions (K>3) were not supported by the program *Admixture*. Pie charts (A and C) and bar plots (B and D) indicate proportions of ancestry for each individual. Subspecies and ecotype belonging of individuals are indicated to the right of the bar plots: BG refers to the Barren-ground subspecies; NM, CM and Boreal refer to the Northern Mountain, Central Mountain and Boreal ecotypes, respectively, within the Woodland subspecies. For NM individuals we also indicate their location in either Yukon (NM-Y, further North), or British Columbia (NM-B) provinces. Basemap layer available from https://www12.statcan.gc.ca/census-recensement/2011/geo/bound-limit/bound-limit-eng.cfm and https://international.ipums.org/international/gis.shtml.

**Fig 3:**
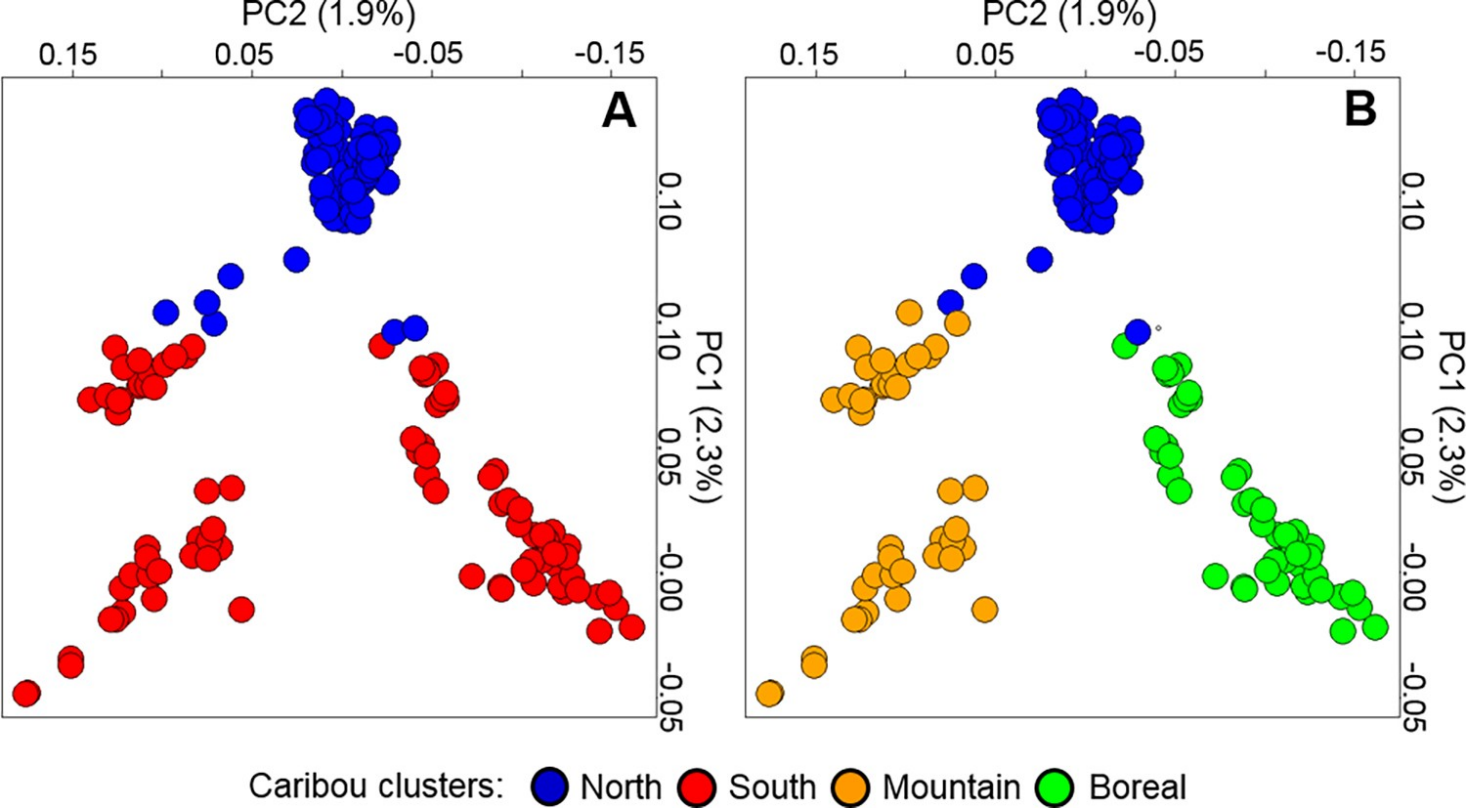
Principal component analysis (PCA) plots of caribou individuals in western North America. Each dot represents a caribou and colors in panels (A) and (B) represent groupings at K = 2 and K = 3, respectively, determined using the program *Admixture* (see Fig 2). PCA was calculated examining 28K SNP data.

We analyzed samples from 190 female caribou, focusing on females because they are fundamental to the management of this endangered species. We identified SNPs using a RAD -seq approach. The dataset comprised 28K SNPs, after excluding linked SNPs and those not in Hardy-Weinberg equilibrium. We then used the maximum likelihood based method implemented by the program *Admixture* for partitioning genetic clusters (K) and found support for at most two populations (K = 2), including a Northern and Southern cluster (mean *Q*_North_ = 0.92, SD = 0.09; mean *Q*_South_ = 0.81; where *Q* = proportion of ancestry) (Fig 2A and 2B, and Fig B in S1 Text). The Northern cluster was formed by individuals (n = 103) of the Barren-ground subspecies and by most Woodland caribou in the Northern Mountain ecotype (those from Yukon). The Southern cluster included Woodland caribou individuals (n = 87) belonging to the Boreal and Central Mountain ecotypes, and caribou of the Northern Mountain ecotype from central-northern British Columbia. The differentiation index *F*_*ST*_ between the Northern and Southern clusters was 0.0171 (C.I. = 0.0166–0.0174). The next partition (K = 3; Fig 2C and 2D) identified a third subdivision consisting mainly of Boreal (n = 42; mean *Q* = 0.87, SD = 0.18) and Central Mountain individuals (n = 43; mean *Q* = 0.85, SD = 0.12). A principal component analysis (PCA), conducted with the program *SmartPCA* within *Eigenstrat*, also revealed genetic separation between North and South cluster caribou along the first axis, with the PCA plots closely resembling the geographic distribution of samples (Fig 3).

### Presence of migratory behavior in each subspecies and ecotype

**Fig 4:**
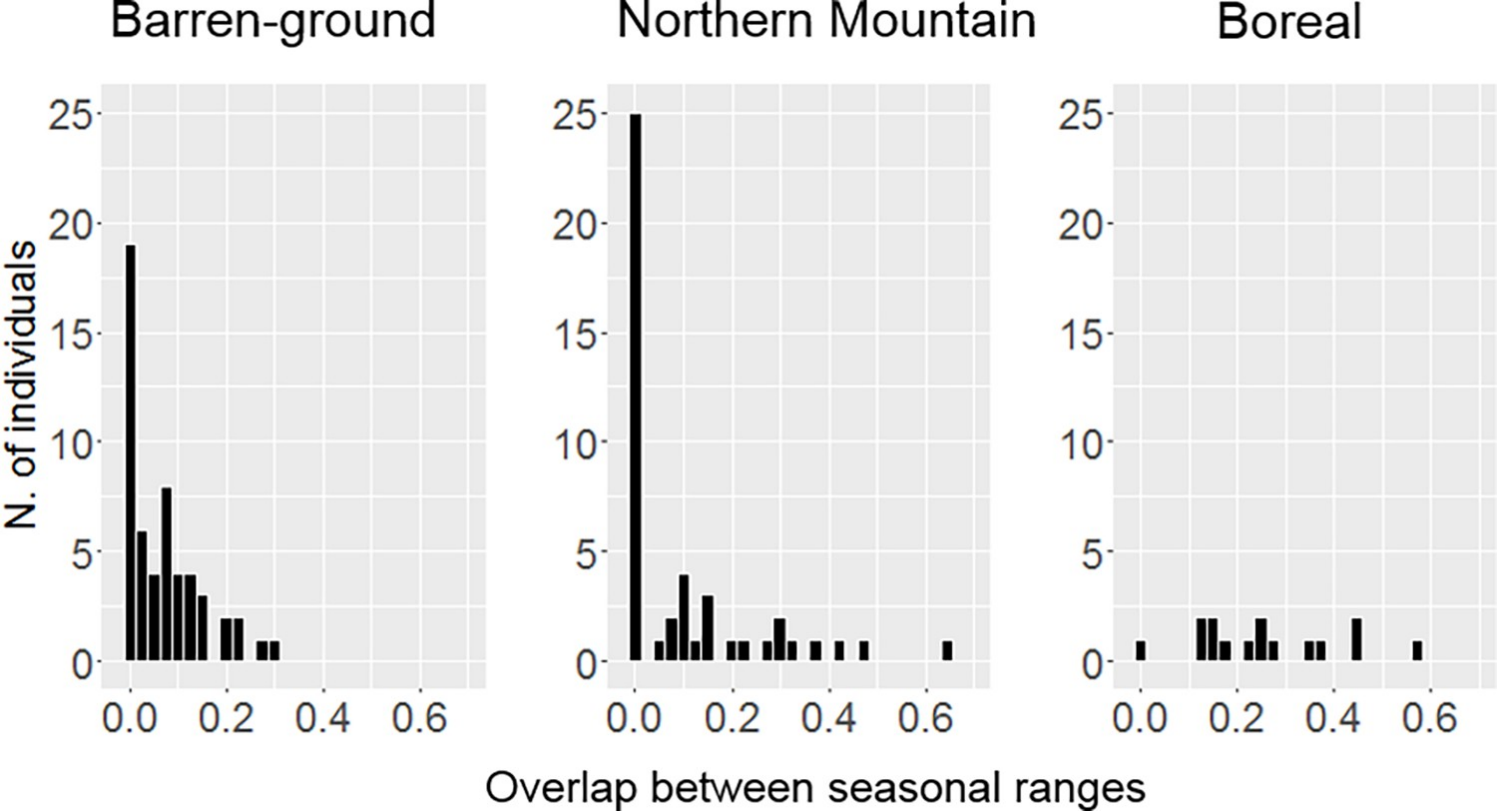
Occurrence of migratory behavior across caribou ecotypes. Histograms represent number of individuals in each ecotype with varying degrees of seasonal ranges overlap. In this study, we correlated genetic traits with this information: sedentary behaviour tendency represented by seasonal ranges overlap—a continuous variable.

**Fig 5:**
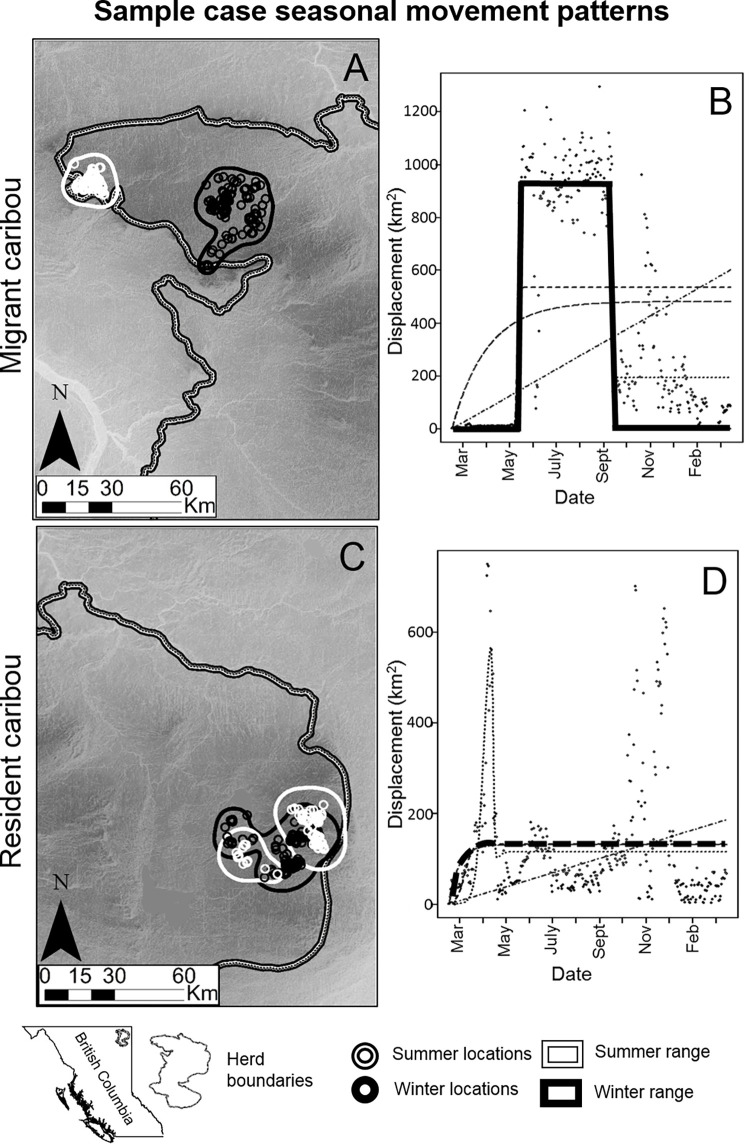
Seasonal movement patterns monitored with GPS-collars for a migratory caribou and for a sympatric resident caribou. (A) and (C) show winter and summer locations. For each caribou (A) shows a complete separation of the seasonal ranges, typical of a migratory animal, while the animal in (C) has ranges overlap and is considered sedentary. (B) and (D) show Net Square Displacement (NSD) plots for the same individuals, between a starting telemetry location and each subsequent location in a year period. (B) The displacement is best represented by a bell curve (marked with continuous line above) when the animal is migratory. (D) An animal is classified as sedentary when the best fitting line quickly reaches an asymptote. Dark grey dots represent actual telemetry locations. Solid lines represent the best fitted models, whereas other lines represent unsupported models of seasonal movement (mixed-migrant, nomad or disperser). In this study, we correlated genetic traits with NSD characterization of individuals as migrants. Basemap layers available from: https://www12.statcan.gc.ca/census-recensement/2011/geo/bound-limit/bound-limit-eng.cfm and https://open.canada.ca/data/en/dataset/957782bf-847c-4644-a757-e383c0057995.

We studied caribou migratory behavior by analyzing telemetry locations of 139 of the 190 females sampled for genetic analysis above (Fig 4 and Table A in S1 Text). For 116 caribou for which there was sufficient location data across seasons, we determined summer and winter ranges with the adehabitHR R movement ecology package. We calculated an index of overlap (IO) between seasonal ranges (higher and lower values indicating resident and migratory behavior, respectively; Fig 5A and 5C). IO for Boreal caribou (mean = 0.29) was greater than that of Northern Mountain (mean = 0.10; pairwise Wilcoxon Rank Sum test, *p*<0.001) and Barren-ground caribou (mean = 0.07; *p*<0.001). In addition, we conducted Net Square Displacement (NSD) analyses with the R package *MigrateR* on the 102 animals that had at least one location data point per day for an entire year of data. Out of these, 83 caribou individuals were classified as migrants (Barren-ground subspecies, n = 44/46; Northern Mountain ecotype, n = 31/40; Boreal ecotype, n = 8/11); remaining 14 caribou were classified as residents using this statistically conservative approach (Figs 5B and 5D); and 5 caribou were classified as either migrants or residents in different years.

We calculated the distance and timing of migration for each caribou, again using the R package *MigrateR*. Average departure dates (when animals started to migrate in the spring) were not significantly different between caribou ecotypes (Kruskal–Wallis test, *p* = 0.16) (Table A in S1 Text). The distance of migration calculated for the Barren-ground caribou (mean = 247.61 km) was significantly greater than for Boreal (mean = 10.31; pairwise Wilcoxon Rank Sum test—*p*<0.001) and Northern Mountain individuals (mean = 42.92 km; *p*<0.001). Migration distances for Northern Mountain caribou were also greater (*p*<0.001) than for Boreal caribou. During spring migration, Barren-ground caribou moved, on average, more northward than other caribou (Northern Mountain, *p*<0.001; Boreal, *p*<0.001).

### Caribou migratory behavior associated with genes involved in brain activity, fat and energy metabolism, body development, and hormones’ production

Using the program *Gemma*, we tested the association between each SNP (a dataset comprising 29K non-linked SNPs) and each of the five measurements of migratory behavior described above: (1) index of seasonal ranges overlap, (2) NSD classification of animals as migratory or resident, as well as (3) distance, (4) departure timing, and (5) latitudinal shift of migration. As is the case for all association studies, associations can be influenced by the demographic history of individuals; therefore, in our regression models we corrected for neutral genetic structure by employing a relatedness matrix that could contribute the genomic inflation factors of 1.33, 1.88, 0.33, 0.24, and 0.37 for IO, NSD classification, and distance, timing, and latitudinal shift of migration, respectively. Genomic inflation factors >1 indicated potential for additional effects of genetic structure on the phenotype. We detected 57 SNPs significantly associated (*p<*0.05 after Bonferroni correction) with migratory propensity in caribou (Table 1 and Table B and Fig C in S1 Text). Three loci were associated with the IO, one of which had the strongest association detected (SNP in gene *UBE3D;* p = 3.35x10^-12^, Fig C in S1 Text); 54 additional loci were instead associated with the binary classification of animals as either sedentary or migratory. The relative contribution of phenotypic variance (*PVE*) explained by each of the SNPs associated with migratory behavior ranged from 1.6 to 34.4% (Table B in S1 Text). Twenty-seven SNPs were located in or potentially linked (within 2 Kb) to 21 genes (n, intron = 21 SNPs, exon = 5, promoter = 1) and their functions included brain activity, fat and energy metabolism, body development, and hormones’ production (Table 1 and Table B in S1 Text; function of genes determined using the program *Ensembl BioMart*).

**Table 1:** Candidate genes associated with migratory behavior in caribou.

SNP position	Gene name	Regulating for	Found associated with/ in	References
intron	*XPNPEP1*	brain activity	behavioral hyperactivity; cognitive deficits/ mouse, horse	[32,33]
intron	*PAK3*		mental disability/human, mouse	[34,35]
intron	*HTRA1*		cognitive impairment; mood disorders/human	[36,37]
intron	*KIF5C*		malformations of cortical development (mental disease)/human, mouse	[38,39]
exon	*DHX30*		sleep disorder/ human	[40]
intron	*ARNTL*	brain activity	Circadian rhythmic expression/fish, mammals, insects, birds	[12,41,42]
intron	*ANO1*		Circadian rhythmic expression/mouse	[43,44]
intron	*PARP1*		Circadian rhythmic expression/human, mouse	[45,46]
intron	***UBE3D***	fat and energy metabolism	variation in fat deposition/sheep cattle	[47,48]
intron	*ATF7*		adipose differentiation/human	[49,50]
intron	*TCF7*		thermoregulation; variation in fat deposit/cow	[51]
intron	*PSD3*		diabetes; obesity/human	[52]
intron	*TMEM163*		diabetes; nutrient sensing/mouse, cattle, human	[53,54]
intron	*MAPKAPK2*		starvation; obesity; diabetes/mouse	[55–57]
exon	*HMCN2*		variation in stimuli response/pig	[58,59]
promoter	*LPIN2*	body development	bone formation; ostiomelite; inflammation of junctures/human	[60,61]
promoter	*THPO*		production of blood cells; body growth	[62,63]
intron	***GPC6***		bone growth; dwarfism/human, cow	[64,65]
intron	*FAM155A*		diverticulitis (variation of intestinal morphology)/human	[66]
intron	***POLR3B***	hormones’ production	hypogonadism/human, mouse	[67,68]
intron	*C14H8orf34*		milk production; age at first parturition/cow	[66,69]

Bold-face gene names are those associated to Index of Overlap. Reported loci were found directly in genes based on annotations of the bovine genome.

We examined pairwise differences in minor allele frequency (*MAF*) between the clusters identified with the Admixture program (above) for the 57 migration-associated SNPs. Differences in *MAF* were found between the North and South clusters (K = 2; Kruskal–Wallis test, *p*<0.001), between the North and Mountain clusters (K = 3; *p*<0.001), and between the Mountain and Boreal clusters (K = 3; *p*<0.001 –Fig D and Table C in S1 Text). A PCA (Fig E in S1 Text) calculated using only the 57 migration-associated SNPs indicated groupings that were less discrete than those obtained with population structure analyses including all SNPs (above).

### Caribou migratory behavior of individuals within ecotypes depended upon ancestry

**Fig 6:**
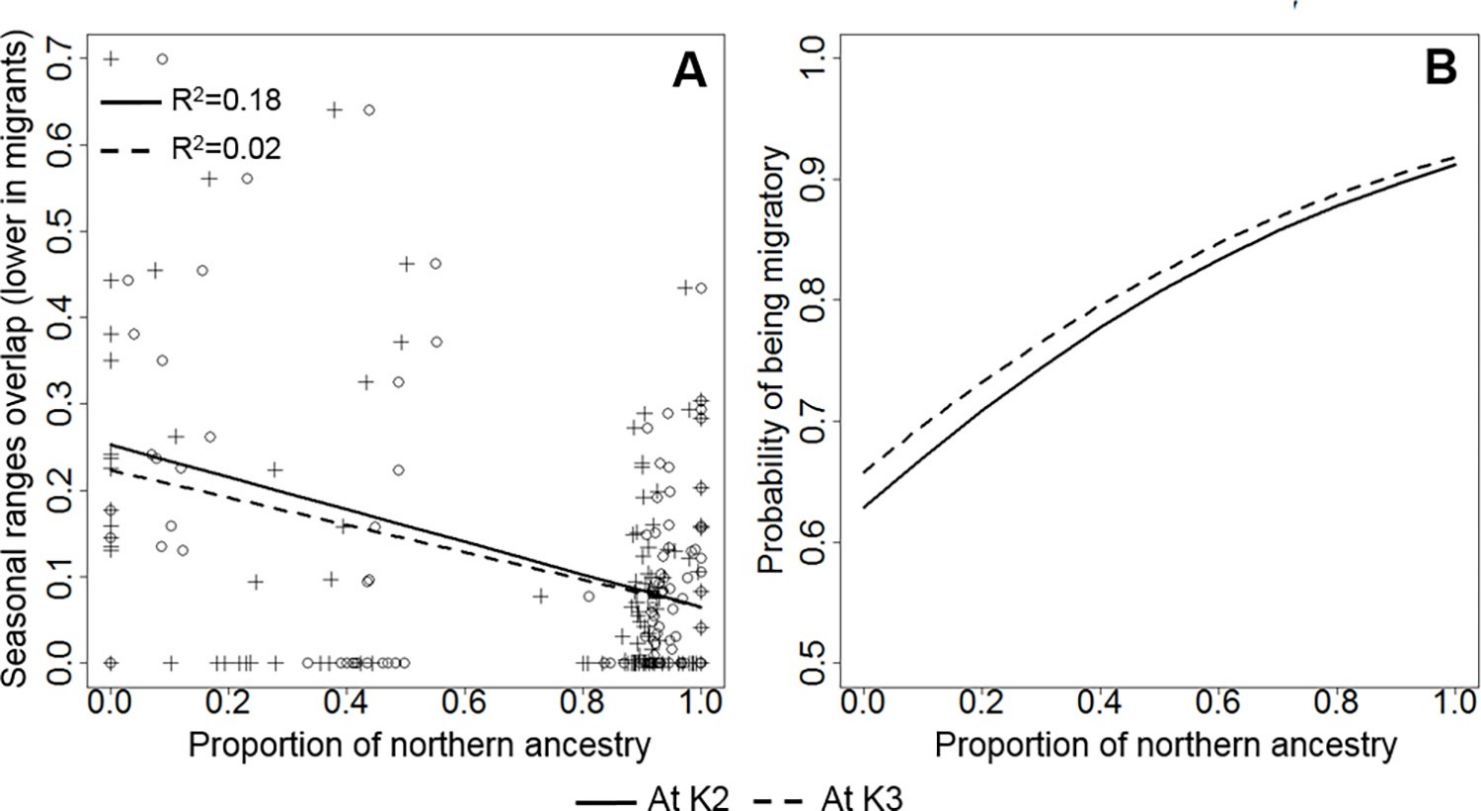
Regression plots between migratory tendency and proportion of genetic ancestry. (A) The overlap of seasonal ranges (Index of Overlap, IO, lower in migrants) was negatively dependent upon an individual’s proportion of assignment (*Q*) to the North cluster, determined using the program *Admixture*. Individual observations at K2 and K3 (i.e. clusters detected) are marked with circles and crosses, respectively. (B) The probability of being migratory as indicated by Net Square Displacement (NSD) was positively dependent upon an individual’s belonging to the North cluster also obtained at both K = 2 (continuous line) and K = 3 (broken line). Panel B includes results of logistic regression, thus indicating a predicted relationship between a genetic trait and migration that is valid for any caribou.

We conducted regression analyses of caribou migratory behavior, as it was evaluated with either ranges overlap or seasonal displacement methods, dependent upon the admixture proportion of ancestries (*Q*). Results indicated that, even when accounting for ecotype differences, sedentary behavior of individuals was influenced by that individual’s proportion of assignment to the Boreal cluster (Table 2). Moreover, when accounting for ecotype differences, migratory behavior was positively associated with an individual’s proportion of assignment to the North cluster (Fig 6 and Table D in S1 Text). Specifically, overlap between summer and winter ranges (IO, an indication of sedentary behavior) was dependent upon *Q* of the Boreal/South cluster obtained at K = 2 (β = -0.19; *p*<0.001; R^2^ = 0.18) and K = 3 (β = 0.29; *p*<0.001; R^2^ = 0.04; accounting for ecotype; Table 2). Similarly, individual migration assessed with NSD depended on *Q* of the North cluster obtained at both K = 2 (β = 1.81; *p* = 0.03; R^2^ = 0.06) and K = 3 (β = 1.69; *p* = 0.04; R^2^ = 0.08; accounting for ecotype; Table D in S1 Text).

**Table 2:** Dependence of individual caribou migration upon ancestry, while statistically controlling for ecotype.

Model (lm(IO) ~	β	Std. Error	t value	Pr(>|t|)	AIC
**Admixture proportion (Q)–K = 2**					
**South**	**0.187**	**0.038**	**4.871**	**3.61E-06**	**-133.35**
*Condition dependence upon ecotype belonging*					
South + (1|ecotype)	0.038	0.065	0.588	0.556	-122.433
**Admixture proportion (Q)–K = 3**					
**Boreal**	**0.229**	**0.037**	**6.258**	**7.05E-09**	**-145.67**
**North**	**-0.158**	**0.035**	**-4.436**	**2.12E-05**	**-129.89**
Mountain	-0.094	0.066	-1.424	0.157	-113.46
*Condition dependence upon ecotype belonging*					
**Boreal + (1|ecotype)**	**0.228**	**0.037**	**6.146**	**7.93E-10**	**-131.835**
North + (1|ecotype)	-0.012	0.055	-0.222	0.824	-121.862
Mountain + (1|ecotype)	-0.107	0.063	-1.698	0.089	-124.96

Results of linear regression analyses examining dependence of overlap between summer and winter ranges (IO, an indication of sedentary behavior) upon admixture proportion (*Q*) for two or three caribou clusters (K); Beta coefficient (β), Standard error, *t* value, *p*-value, and AIC are indicated, while the models in parentheses account for caribou ecotype as a random effect. Models in bold are significant; IO = ranging from 0 (migrant) to 1 (fully sedentary).

## Discussion

We used an integrated approach of ecology and genomics to study the genetic basis and ancestry of migratory behavior in caribou. Here we document the seasonal movements of GPS-collared caribou and, surprisingly, detect the presence of individual migrants in all subspecies and ecotypes. Caribou migratory behavior was found to be associated to genes known to influence migratory propensity in other organisms. We also determined a genetic subdivision of caribou into a Northern and a Southern genetic cluster, with individuals possessing varying degrees of such ancestries. Finally, our findings indicate that the propensity to migrate depends upon the proportion of ancestry in individual caribou, therefore suggesting a genomic legacy of migration in endangered caribou.

### Genes influencing migratory behavior in caribou: A package common in other migratory species?

We identified an association between individual variation of migratory behavior and genotype variation in a large terrestrial mammal: the caribou of western North America. Here we determine genetic mutations (i.e. SNPs) associated with migration detected via GPS collars in caribou, by using an integrated approach involving technologies that are best suited to describe migratory behavior. Some of the associated SNPs were intergenic, likely noncoding DNA and therefore not involved in functions related to migration (Table B in S1 Text). Alternatively, these SNPs were tagging other, unobserved SNPs that may be involved in migration. Because our RAD-seq approach only covers a minor portion of the genome, additional SNPs that were not assessed in this study may still be associated with the migration phenotype. However, some of our associated SNPs were found in genes with known or hypothesized mechanistic roles in determining migration in other species, including circadian genes, genes involved in sleep and cognitive disorders, and genes regulating fat metabolism and hormone’s production (Table 1). Finally, propensity to migrate depended upon the proportion of Northern or Southern ancestry in individual caribou, and thus on the evolutionary history of its migratory and sedentary subspecies [70] dating back to the last glaciation. Overall, our findings provide initial evidence of an ancestral genes’ package common across migratory taxa that affects the propensity to migrate.

Our study indicates that genes involved in brain activity could contribute to migration. In particular, some migration-associated mutations were found in circadian genes (*ARNTL*, *ANO1*, and *PARP1*), perhaps indicating that the timing of seasonal movements might be genetically influenced. Our findings therefore support the notion that animals synchronize their movements during migration with the seasonal availability of necessary resources [9], and migratory animals may keep track of the seasons using an endogenous timer [12]. Other studies of caribou have claimed that the initiation of migration is influenced by environmental conditions [28,71]; however, these may interact with genetics, as reported in other migratory species [8,16]. Involvement of *ARNTL*, *ANO1*, and *PARP1* in migratory behavior and seasonality has been reported in other species of mammals, fishes, and birds [43,72], and *ARNTL* in particular has been shown to play a particularly major role [12,42].

Some migration-associated mutations were found in other brain activity genes involved in sleep and cognitive disorders in mice and humans (prominently *XPNPEP1* and *PAK3*). Numerous migratory animals, including the caribou we monitored in this study, perform long-distance migrations with only a few resting stops [9]. Therefore, migration might proceed at a pace that does not allow migratory animals much time for sleep or rest, and the mutations we detected may contribute to preserving cognitive and physical performance while migrating [73].

Other migration-associated mutations were found in genes determining obesity and diabetes in human, mice, sheep, and cattle (e.g. gene *UBE3D* and *TCF7*). This may indicate that, thanks to specific genetic mutations, migratory caribou could enhance metabolization of fat and carbohydrates to fuel energetically expensive migration. Migration is particularly energy-demanding [9], so mutations such as those we documented could be beneficial [74,75]. In particular, *UBE3D* and *TCF7* control the metabolism of fat—a primary fuel for migratory animals [8,76].

In migratory caribou, we also documented mutations in genes involved in hormone production, suggesting their potential role in regulating the timing of migration. The *POLR3B* gene (associated with seasonal ranges overlap) regulates the sexual hormone *GnRH*, which in turn has been found to control the timing of migration in fishes and birds [77,78]. Finally, gene *C14H8orf34*, which was also associated with migration in this study’s caribou, is known to determine parturition timing in cattle [66]. Therefore, our findings indicate a potential role of genetics in the timing of caribou parturition, which is temporally correlated to the end of migration (Bergerud et al. [27]).

### Surprising presence of migrants in all caribou subspecies and ecotypes

Using genomics, this study confirms previously reported aspects of caribou taxonomy in western North America, since the two clusters we describe tend to correspond to currently known subspecies, as determined with autosomal microsatellites [24,79]. However, we detect a boundary between the two subspecies that is farther South compared to previous work that used neutral markers, and largely corresponds to other works using genomics, but at a coarser scale [80,81]. In addition, the three sub-clusters that we detected correspond to the Barren-ground subspecies, Boreal ecotype, and Central Mountain ecotype, with a clear hybrid zone of Northern Mountain caribou characterized by assignment to all three sub-clusters. Population structure analyses that were based only on migration-associated SNPs indicated groupings that were less discrete, suggesting that at least some of the migration-associated loci may be under balancing selection and therefore less differentiated between populations, although the matter is debated in the literature [82]. Consistent with this interpretation, Cavedon et al. [80] detected signals of balancing selection in this study area’s caribou. Regardless of the discrepancies, our approach is not designed to reassess caribou taxonomy, as this should depend on the integration of multiple factors, not just genomics [83].

This study’s Barren-ground caribou exhibit long-distance migrations (one way mean = 247.61 km; range = 77.96–467.36), as expected in this species that is arguably characterized by “the longest terrestrial migrations and movements around the world” [84]. For Woodland caribou, we also detect migrations, although their distances are dramatically shorter (> tenfold). Ultimately, we identify migratory individuals across all studied caribou subspecies and ecotypes, despite the Woodland subspecies being previously described as largely sedentary (but see [85]). This study’s collaring of females only was decided by the governing bodies, as these were considered as a first monitoring priority for conservation. Females are also ideal for defining seasonal movements in caribou, as they show fidelity to areas used during a fixed calving period occurring each year. In caribou, also migrate, likely in equal proportion to females [86]. However, future studies could be conducted to look at the migration patterns and genetic influences in males in particular.

Consistent with the literature, we detected more sedentary animals and less migratory animals within the Woodland subspecies, and particularly within the Boreal ecotype [27]. Boreal individuals also had larger overlap between their summer and winter ranges, indicating a higher tendency to be sedentary. However, some migratory and sedentary animals were detected in our study within populations of the Boreal ecotype and Barren-ground subspecies, respectively. Therefore, our data indicate partial migration (*sensu* [19]) in populations belonging not only to Mountain caribou (where this population trait was known [24]), but also to the Barren-ground subspecies and Boreal ecotype. Overall, we detected a large proportion of migratory individuals across all subspecies and ecotypes and in populations where individuals are likely exposed to the same environment. Sympatry of migrants and residents suggests that the difference between the two behaviors could be genetically rather than environmentally caused. As an alternative explanation, other factors may also influence caribou migration, including differences in age, experience, and sex (not accounted for here as we only monitored females), among others. As a result, migration can be obligate (for example, genetically determined) or facultative within the same population even if individuals all experience the same environment [3].

### Mechanisms of migration: Intrinsic genetic forces and the role of ancestry

Overall, the results of our association analyses indicate that genetic mutations could produce two opposite phenotypes in caribou: migratory or resident individuals. As explained by Cavedon et al. [80], the existence of both migratory and resident caribou in sympatry could be promoted by negative frequency-dependent selection, which is a form of balancing selection allowing the existence of multiple haplotypes and phenotypes within populations (note that the study relied on pooled samples and could not conduct individual analyses as in this study). Phenotypical “bimodality” in caribou, as described for example by Cavedon et al. [80], contrasts with other migratory species, including other cervids. For instance, elk are behaviorally plastic and exhibit wide variation in their migratory behavior across their lifetimes [87]. Consistent with bimodality, our study’s SNPs were not associated with continuous migratory patterns, within the migratory form, including distance, timing, and latitudinal shift of migration, which are under genetic control in other species (see for example [88]).

Our findings suggest the presence of intrinsic genetic drivers of migration and are consistent with recent work by Gurarie et al. 2019 [28] examining caribou migration with GPS location data. The authors observed synchrony of animal migratory movement even if individuals belonged to different populations, suggesting an intrinsic determination. This intrinsic drive can arise from a response to common cues (i.e. day length), but may also be promoted by genetic mutations, such as the ones we observed (above). However, in this study we also document that a few caribou individuals (5/102) are migrants or residents in different years; this indicates the existence of some seasonal movement plasticity in the species. These switches in behavior could be attributed to environmental changes from year to year, to learning, promoted by some genetic traits, or a combination of all the above.

Our results also indicate that the differences between the migratory and sedentary type could be related to the long-term evolutionary history of caribou, a species that during the last glaciation evolved into two separate subspecies, north and south of the continental ice-sheet, respectively [29]. Our population structure analyses indicates a main North-South separation of caribou, and confirms the presence of two lineages [24,79]. In our study, individual propensity to migrate depended upon the proportion of assignment to the North cluster, as determined by the *Admixture* ancestry analysis. Correlation coefficients were significant, but their values were not substantial. Therefore, our correlations might not indicate a simple cause-to-effect relationship between ancestry and migration. It is also possible that our correlations were weakened by uncertainties, including uncertainties in *Q* value estimates of ancestry, as well as those in our determinations of migration.

Despite the uncertainties, all our analyses were conducted while statistically controlling for ecotype differences, therefore indicating ancestry effects detectable in caribou individuals also belonging to the same ecotype. These results are strongest (i.e. 18% of variance explained) when we used the seasonal ranges overlap as the variable representing migratory behavior. Thus, individuals with northern-type DNA and the associated “ancestral” genes could be more prone to migration than those without. Overall, an ancestral gene-to-behavior association—that was likely advantageous for tracking seasonal resources in the tundra and taiga (regions frequented by the northern subspecies during glacial times [70])—is likely retained in some individuals throughout our study area. After glaciation, caribou recolonized vast areas that were previously covered by continental ice, also including the mountainous parts of our study area [24]. Perhaps aided by northern ancestral genes, caribou are presently capable of migrating either from tundra to taiga areas in the North (as in glacial times too), or from alpine to forested areas in the mountains.

### Conservation implications: Potential extirpation of migrants

As a result of habitat alteration caused by anthropogenic activities (including barriers), dramatic declines in populations of migratory ungulates and the disappearance of migratory behavior are now recognized as a global conservation challenge [89,90], with alarming new findings for threatened caribou, in particular [31].

Human-caused habitat alterations and climate change are both implicated in caribou decline [31,91] and, together with the extirpation of some populations, ecological and genetic traits could also be extirpated in the future. We document that caribou migration and associated genes are unequally distributed among subspecies, ecotypes, and populations. If, as we report, migratory behavior is genetically influenced, caribou could be further impacted, possibly by permanent loss of the migratory trait in some populations already at low numbers [26,30]. These results also suggest that the migratory trait, and the set of mutations contributing to the trait could not be easily re-established when lost in a population. Recent findings confirm that migration is imperiled in endangered caribou populations [31]. Genetic mutations, especially those that are beneficial, occur in evolutionary timeframes [92], perhaps incompatible with the fast decline of caribou. In the face of rapid declines, novel mutations, including those influencing migration, are unlikely to emerge on time. This loss could perhaps be averted with the maintenance of critical seasonal habitats (*sensu* [93,94]) for caribou within and between seasonal ranges–a strategy also allowing for long-range movements and migration.

We believe that our concerns for the loss of migration in caribou are transferable to other species and systems where there are documented declines, and migration is likewise associated with genes. The loss of migration can have significant ecological impacts on ecosystems, such as influencing prey densities and grazing in seasonal ranges, so understanding the mechanisms underlying migratory behavior in ungulates has become a broader conservation priority [22,89,95,96]. Our study on the drivers of migration in caribou is therefore applicable to the management and conservation of wild migratory ungulates in general, as well as their environments. In other ungulates too the proportion of genetically-enabled migrants in populations might be declining. As a consequence, fewer and fewer migrants could perform their ecological role in the future. Finally, our study reveals that caribou possess a gene package common to other migratory species. These genes were found to not only be associated with migration, but also with other ecological, morphological, and behavioral traits of adaptive value in model and non-model species. Thus, the potential loss of genetic mutations influencing migration may also result in the loss of other important traits; conversely, preserving these mutations could maintain a whole suite of traits promoting a species’ survival in the long-term.

## Material and methods

### Ethics statement

Research was conducted under research permits of Government of British Columbia, Alberta, Northwest Territories, and Yukon, Parks Canada, University of Calgary, and University of Montana. Approval was granted by the University of Calgary’s Life & Environmental Sciences Animal Care Committee (LESACC), ACC Study #AC16-0195.

### Samples collection and molecular analyses

Blood and tissue samples from 284 female caribou (note below rationale for pick of sex) were obtained from monitoring activities across western North America from 2004–2016. Sampled caribou were from two recognized subspecies and three ecotypes: 60 individuals belonged to the Barren-ground subspecies (*R*. *t*. *groenlandicus–*also forming its own ecotype) and 224 individuals belonged to three ecotypes within the Woodland subspecies (*R*. *t*. *caribou;* Boreal, n_individuals_ = 96; Northern Mountain, n_individuals_ = 99; Central Mountain, n_individuals_ = 29) (Fig 1). In this study, we examined caribou belonging to three ecotypes (Boreal, Central Mountain, and Northern Mountain) within the Woodland subspecies (*R*. *t*. *caribou*).

We extracted DNA from samples with the DNeasy Blood and Tissue Kit (*Qiagen*) following manufacturers’ protocols and identified high-quality DNA as a high molecular weight band (>1 Kb) on a 2% agarose gel with a 2-log DNA ladder. We subsequently quantified DNA using either *PicoGreen* or *Qubit* 2.0 fluorometry and standardized it to a final concentration of 5 ng/μL per sample. DNA was digested with the *SbfI* restriction enzyme to prepare restriction site associated DNA sequencing (RADseq) libraries, barcoding each individual sample, following Ali et al. [97]. Unique barcode tags allowed us to 96 samples into a single genomic library, without losing track of individual data. The genomic libraries were then sequenced with paired-end 2x100nt reads on an Illumina HiSeq 2500 at Princeton University’s Lewis-Sigler Institute for Integrative Genomics core facility (full details in Method A in S1 Text).

### RADseq analysis and SNPs finding

Using a custom *Perl* script, we filtered raw sequencing reads to retain only those that contained the *SbfI* cut site, along with a barcode. We further demultiplexed (obtained reads for each individual) and filtered reads using the *process_radtags* and the *clone_filter* scripts within *Stacks* v2.0 [98]. We retained individuals with a minimum of 500,000 reads [99] and these reads were then mapped to the reference *Bos Taurus* genome (UMD3.1- [100]) using *Stampy* v1.0.20 [101]. Our pick of the *Bos Taurus* genome was motivated by both its quality and established used in the literature for cervids (see for example [102]) like caribou. We then used *SAMtools* v1.5 to remove reads with low mapping quality (MAPQ < 60) and to obtain files, in BAM format, for each individual [103]. To discover SNPs, we used the BAM files within *Stacks* 2.0. We therefore ran the *gstacks* and *populations* modules, with the latter implemented twice (see details on SNPs calls in Results A in S1 Text). In the first implementation, we retrieved only loci that were genotyped in 90% of individuals and had a minor allele frequency greater than 0.05. Loci obtained with this first run were examined with *VCFtools*, which was used to calculate the number of SNPs and the total missingness (number of missing SNPs) per individual [104]. In the second implementation of *populations*, we used previous filtering options and additionally we removed individuals with >85% of missingness (i.e. individuals with very high proportion of missing SNPs) and/or with mean coverage < 3x. Ultimately, we retained 190 individuals (out of 284), which belonged to two recognized subspecies and three ecotypes (Fig 1A): 57 individuals belonged to the Barren-ground subspecies and 133 individuals belonged to three ecotypes within the Woodland subspecies (Boreal, n_individuals_ = 34; Northern Mountain, n_individuals_ = 76; Central Mountain, n_individuals_ = 23) (Fig 1A). Source data for all caribou before and after filtering are provided as S1 Data. Furthermore, we ran *populations* to retrieve only the first SNP per locus. We therefore discovered 31,080 SNP loci, which were further filtered for linkage disequilibrium (referred to as the “29K LD SNPs set”) in *Plink* v1.9 (flag “—indep-pairwise 50 5 0.5”–[105]).We finally filtered the LD SNPs set to retain those SNPs in Hardy-Weinberg Equilibrium (flag “—hwe 0.001” in *Plink*). The expectation was that this SNP set contains putatively neutral loci useful for population genetic and demographic analyses, hereafter referred to as the “28K neutral SNP set”. For further analyses with migration, the SNP datasets were used for this study’s 139 GPS-collared caribou (see below).

### Population structure analyses

To visualize the genetic structure of our individuals, we performed a principal component analysis (PCA) using *SmartPCA* within *Eigenstrat* v3.0 [106] for the 28K neutral SNP set. Next, we evaluated genetic structure using a maximum likelihood approach implemented in *Admixture* v1.3 with the 5-fold cross-validation flag for K varying from 1 to 22 (number of sampled caribou herds) to capture the fine population structure of caribou [107]. Assignment of individuals to specific clusters was obtained following Schweizer et al. [108].

### Assessment of migratory behavior in caribou

#### Data collection and screening

Female caribou were radio-collared by government staff or contractors of Yukon, British Columbia, Northwest Territories, and Alberta between 2004–2016, each following their respective government’s standardized permitting, animal care, and handling procedures. Collars varied with respect to their duration on caribou (minimum = 2 months, maximum = 6 years) and were equipped with a fix interval (e.g. number of locations per day) ranging from hourly to every 7 days. We therefore filtered and standardized telemetry data for each animal to obtain a maximum number of daily locations equal to one. After screening procedures, the data set contained 75,223 locations from 139 unique individuals: 54 individuals belonged to the Barren-ground subspecies, and 85 individuals belonged to two ecotypes within the Woodland subspecies (Boreal, n_individuals_ = 18; Northern Mountain, n_individuals_ = 67).

#### Determining migration with overlap analysis of seasonal ranges

We excluded caribou individuals with <1 year of monitoring. We then calculated an index of overlap (IO) between winter and summer ranges frequented by individual caribou, with IO ranging from 0 to 1 (higher and lower values indicating resident and migratory behavior, respectively).

To calculate IO, we defined summer (1 July—15 September) and winter (1 December- 30 April) seasons following [24] and we used only individuals with at least 30 locations per season (n = 116) [109]. For each animal we estimated seasonal utilization distributions (UD) using the *kernelUD* function (using reference bandwidth) within the *adehabitHR* package [110] in R version 3.5. We then derived range contour polygons from the 95% fixed-kernel isopleth. Finally, we determined the IO between summer and winter polygons for each animal following [24]:

$$\mathrm{IO}=[2\;A_{12}/(A_{1}+A_{2})]$$

Where *A*_12_ is the area of overlap (km^2^) between the summer and winter 95% isopleths, and *A*_1_ and *A*_2_ are the areas (km^2^) of the summer and winter 95% isopleths for the animal, respectively. For animals with multiple years of data, we averaged IO across years.

#### Individual classification as either migrant or resident

We classified each caribou as either migratory or sedentary by conducting Net Square Displacement (NSD) analyses with the *R* package *MigrateR* [111], which examine seasonal displacement of individuals by fitting trajectory lines (sequences of GPS points) to annual telemetry locations. The best fitting trajectory line was selected, by *MigrateR*, with the Akaike Information Criteria. To improve model fitting, we used the Relative Net Squared Displacement option (rNSD), which allows one to manually set the parameter ρ. This parameter (ρ) defines the minimum number of days that an animal must spend in a second range in order to be considered migratory. We set ρ equal to 30 (therefore, a caribou needed to spent at least 30 days in a second range to be considered migratory) following recommendations in recent publications reporting NSD analyses applied to ungulates [112,113]. We only included caribou with at least one continuous year of data (n = 102 individuals) and when the same individual had multiple years of data, we used it in subsequent analyses only when consistently classified as either migratory or sedentary across years (n = 83 and n = 14 individuals, respectively). Individual animals may not be limited to one seasonal movement behavior throughout their lifetime. Instead, they may demonstrate more than one movement behavior by switching behaviors between years, exhibiting behavioral plasticity (though this is undocumented for caribou). In this study, plasticity in seasonal movements was detected in only a few individuals (n = 5), and these were not used for analyses that were naturally designed to pick a signal of association with genetic traits that are fixed for life (below). These five individuals had a mean of 2.8 years of data (range 2–4), and during these periods the mean number of switches per individual was 1.6 (range 1–4), which contrasted to the lack of any switches in the additional 97 caribou that we also analysed (1.7 years of data per individual, range 1–4).

#### Variation in migratory patterns (distance, timing, and latitudinal shift)

Also with the R package *MigrateR*, we selected individuals consistently classified as migratory (see above), and obtained the parameters δ and t representing the distance separating seasonal ranges and the start date of migration, respectively. Units of migration timing were calendar dates (e.g. 1 January 2017), which we subsequently standardized to numeric dates from a starting day (day 1 = 1 January). In this way, for example, a migration starting on calendar day April 10^th^ was converted to the numeric date 100 (i.e. 100 days after day one). Whenever the same animal had multiple years of data, we averaged numeric dates and the distance of migration across years.

In addition, for each animal, we calculated centroids of winter and summer ranges and calculated the Euclidean distances between these two points. Finally, we used the geographic coordinates of the two centroids to calculate differences in latitudes between winter and summer ranges (latitudinal shift).

### Association study between SNPs and migratory behavior

We ran a univariate mixed model with the software package *Gemma* [114] to examine the dependence (or association) of migratory behavior upon each single SNP. Such associations were tested with each of the five measurements of migratory behavior described above: (1) index of seasonal ranges overlap, (2) NSD classification of animals as migratory or resident, and (3) distance, (4) departure timing, and (5) latitudinal shift of migration. RADseq data, with coverage equal to or lower than that obtained in this study (see above), has been shown to successfully detect trait associations [115]. Following guidelines [114], we accounted for population stratification. We therefore first used the 29K LD SNP set to calculate the relatedness matrix of caribou individuals, and then we incorporated the matrix, as a covariate, in the mixed model. We used Wald’s test to determine the significance of our analyses, where a SNP was considered associated with migratory behavior only when the Bonferroni adjusted *p* value was below 0.05. Ultimately, we calculated the proportion of variance in phenotype (i.e. migratory behavior) explained (*PVE*) by a given associated SNP as described in Shim et al. [116]:

$$PVE=\frac{2\beta^{2}*MAF*(1-MAF)}{2\beta^{2}*MAF*(1-MAF)+(se({\beta ))}^{2}*2N*MAF*(1-MAF)}$$

Where *β* is the regression coefficient obtained with *Gemma* analyses (above), *se* is the standard error of *β*, *MAF* is the minor allele frequency, and *N* is the sample size. When we calculated *PVE* values for all SNPs associated to NSD’s classification as migratory (while in other studies only some are reported; [117]), a theoretical total variance >100% was explained; we therefore provided values for the relative contribution of phenotypic variance (*PVE*) explained by each of the associated SNPs, imposing a total sum of 100%.

We annotated all SNPs as genic (intron or exon), as within a promoter (i.e. we considered any gene within 2 Kb), or as intergenic using an in-house python script [118]. For genic SNPs, we inferred gene functions of those associated to migratory behavior using *Ensembl BioMart* [119].

### Predicting migration dependent upon ancestry

We tested the probability that an individual caribou was migratory dependent upon its ancestry by conducting regression analyses. Our independent variable was the proportion of ancestry of a given individual (a *Q* value of its belonging to a specify cluster, one at a time) obtained with the *Admixture* program (above); for our dependent variable, we used either IO (seasonal ranges overlap) or NSD’s classification of migratory vs. resident behavior. For the first analysis, we used a linear regression, since IO was a continuous variable. For the second analysis, we used logistic regression with an output of either 1 or 0 (migratory or sedentary). Additionally, we tested migration depending upon *Q* while also statistically controlling for ecotype. For these additional analyses, we used the *glmer* function within the *lme4* package in R, where ecotypes were used as random effects [120].

## Supporting information

S1 Text Supporting Methods, Results, Figures and Tables. Fig A. Migratory patterns of caribou sampled in western North America. Fig B. Cross Validation (CV) plot obtained with the Admixture program. Fig C. Manhattan plots of SNPs associated to migratory behavior. Fig D. Differences in minor allele frequencies between caribou genetic clusters. Fig E. Principal component analysis (PCA) plots of caribou individuals based on migration-associated SNPs. Table A. Metrixes of migratory behavior in caribou ecotypes. Table B. SNPs associated to migratory behavior in caribou. Table C. Differentiation of minor allele frequencies (MAF) between caribou genetic clusters (K) detected with the Admixture program. Table D. Dependence of individual caribou classification as migrant upon ancestry, while statistically controlling for ecotype.
(DOCX)

S1 Data Attribute information for caribou samples collected in western North America. (XLSX)
