# Supplementary material for: Genomic legacy of migration in endangered caribou
Source: PLoS Genet. 2022 Feb 10;18(2):e1009974. doi: 10.1371/journal.pgen.1009974 (PMC8830729; doi:10.1371/journal.pgen.1009974)
Supplement: S1 Text — Fig A. Migratory patterns of caribou sampled in western North America. Fig B. Cross Validation (CV) plot obtained with the Admixture program. Fig C. Manhattan plots of SNPs associated to migratory behavior. Fig D. Differences in minor allele frequencies between caribou genetic clusters. Fig E. Principal component analysis (PCA) plots of caribou individuals based on migration-associated SNPs. Table A. Metrixes of migratory behavior in caribou ecotypes. Table B. SNPs associated to migratory behavior in caribou. Table C. Differentiation of minor allele frequencies (MAF) between caribou genetic clusters (K) detected with the Admixture program. Table D. Dependence of individual caribou classification as migrant upon ancestry, while statistically controlling for ecotype. (DOCX) [file pgen.1009974.s001.docx]

**Genomic legacy of migration in endangered caribou**

**S1 Text, Supporting Information**

**Method A. RAD protocol for library preparation.**

We followed the modified restriction site associated DNA sequencing (RADseq) protocol [1] to prepare genomic libraries for 308 caribou from north western North America. Genomic DNA samples were digested with *sbfI* followed by ligation of a unique 8 bp-barcoded biotinylated adapter. We pooled 96–308 samples (in some cases, the same sample was included multiple times in the same library and also in multiple libraries to improve sequencing coverage), which were then randomly sheared to 400 bp on a Covaris LE220. We enriched for adapter ligated fragments using a Dynabeads M-280 streptavidin bead binding assay (Thermo Fisher Scientific). We then prepared final genomic libraries using either the NEBnext Ultra DNA Library Prep Kit or the NEBnext UltraII DNA Library Prep Kit as per the manufacturer’s instructions (New England Biolabs, Ipswich, MA, USA). We selected for genomic fragments between 300–400 bp in size using Agencourt AMPure XP magnetic beads. We then standardized libraries to 10nM, which were then paired-end sequenced (2 x 150 nt) on two lanes of the Illumina HiSeq 2500 at Princeton University’s Lewis-Sigler Institute for Integrative Genomics core facility.

**Results A. Details on SNPs analyzed before LD and HWE filtering**

We identified SNPs by using *gstacks* and *populations* modules within *Stacks 2.*0 [2]. We run the *populations* modules twice. In the first implementation, we retained 50,071 SNPs that were genotyped in 90% of individuals and had a minor allele frequency greater than 0.05. We conducted an initial filtering of this dataset (i.e. we removed individuals with >85% of missingness) and then we ran a second implementation of *populations*, retrieving only the first SNP per locus. We therefore obtained 31,080 SNPs before conducting further filtering (LD and HWE).

**Fig A. Migratory patterns of caribou sampled in western North America.**


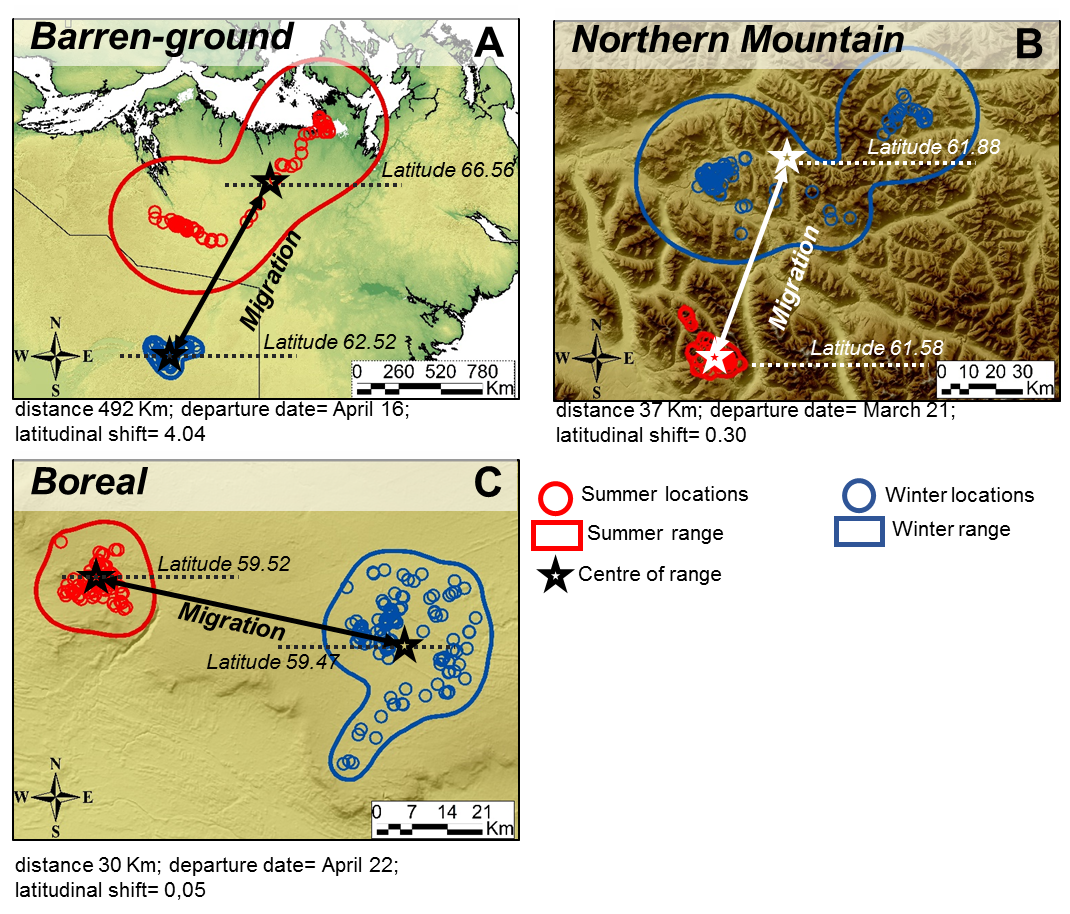


**Fig A.** **Migratory patterns of caribou sampled in western North America**. Winter and summer ranges, and latitudes of their centroids, for three sample caribou belonging to Barren-ground (A), Northern Mountain (B) or Boreal ecotype (C), respectively. Distances between seasonal ranges, departure timing and latitudinal shifts of migration are reported. Basemap layers available from:

<https://www12.statcan.gc.ca/census-recensement/2011/geo/bound-limit/bound-limit-eng.cfm>

and

<https://open.canada.ca/data/en/dataset/957782bf-847c-4644-a757-e383c0057995>

**Fig B. Cross Validation (CV) plot obtained with the *Admixture* program.**

**Fig C. Cross Validation (CV) plot obtained with the *Admixture* program.** CV plot showed maximum support for K (number of ancestral populations) = 2, followed by K=3.

**Fig C. Manhattan plots of SNPs associated to migratory behavior.**

*
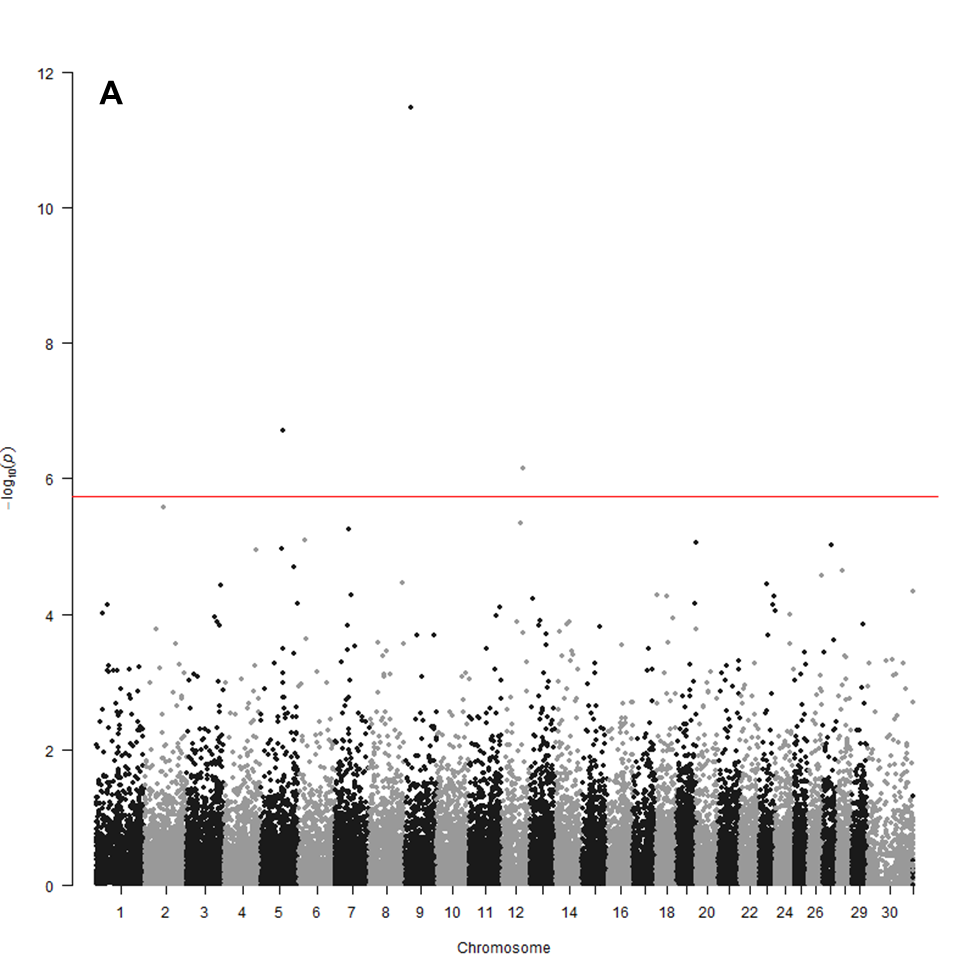
*


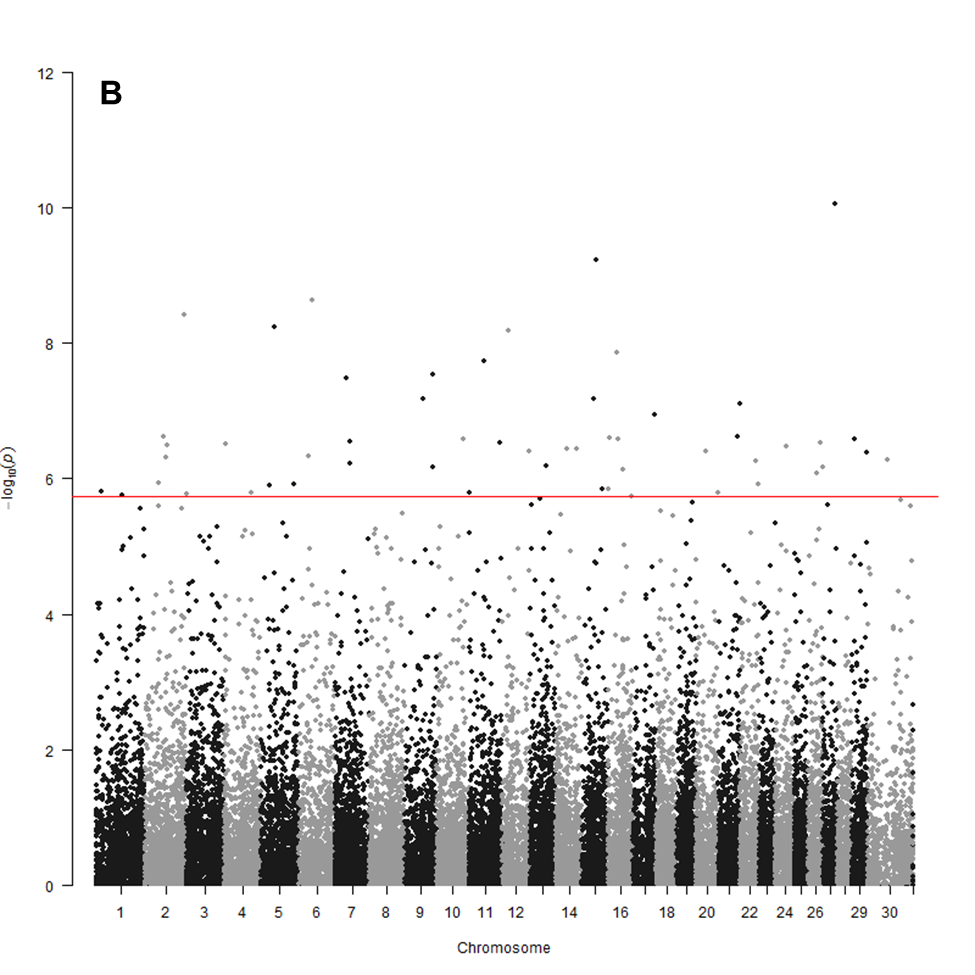


**Fig C. Manhattan plots of SNPs associated to migratory behavior.** (A) shows SNPs associated to the index of overlap between winter and summer ranges frequented by individual caribou. (B) shows SNPs associated with to binary classification of animals as either migratory or sedentary. Chromosomes are on the x-axis and the –log 10 of the p-value is on the y-axis. The red line represents the threshold for significance after Bonferroni correction with variants above this line significantly associated. Chromosomal information and position of loci were based on mapping to the cow genome

**Fig D. Differences in minor allele frequencies between caribou genetic clusters.**


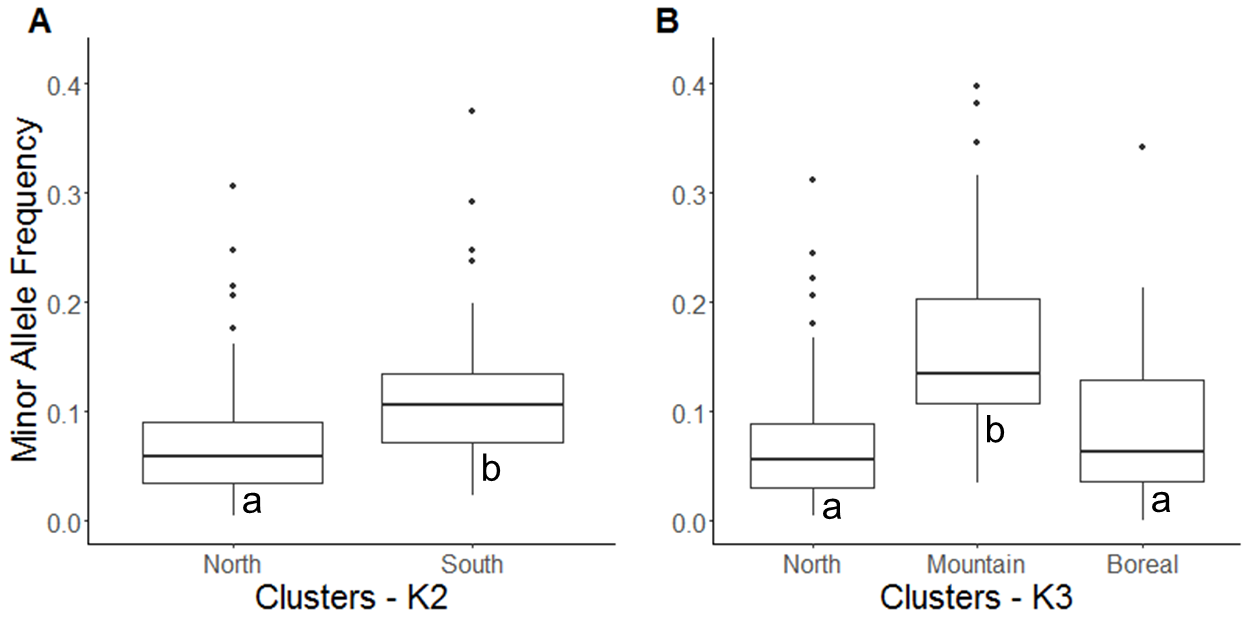


**Fig D. Differences in minor allele frequencies between the caribou genetic clusters.** (A) Differences between North and South genetic clusters detected with the *Admixture* program at K=2. (B) Differences between North, Mountain and Boreal clusters detected at K=3. The lower-case letters a/b and their combinations denote significant differences. Overall differentiation indices *Fst* calculated on all SNPs, for K=2 are reported in Results. At K=3, the *F_ST_* between the Northern and Mountain clusters was 0.026 (C.I.= 0.025-0.027); whereas the *F_ST_* between the Northern and Boreal clusters was 0.026 (C.I.= 0.025-0.028). The *F_ST_* between the Mountain and Boreal clusters was 0.030 (C.I.= 0.029-0.031).

**Fig E. Principal component analysis (PCA) plots of caribou individuals based on migration-associated SNPs.**


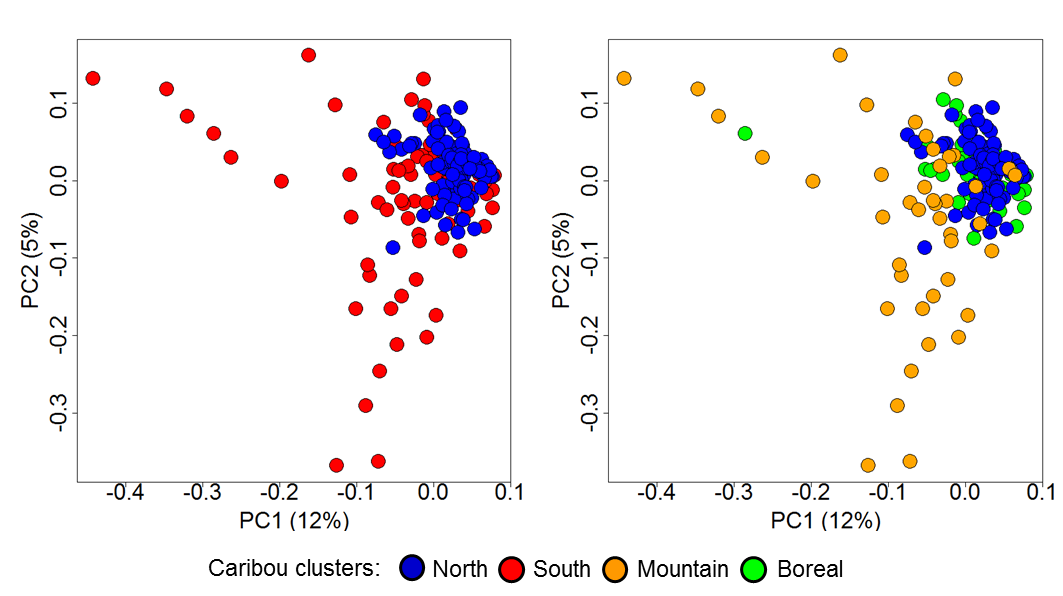


**Fig E. Principal component analysis (PCA) plots of caribou individuals based on migration-associated SNPs.** Each dot represents a caribou and colors in panels (A) and (B) represent groupings at K=2 and K=3, respectively, determined using the program *Admixture* (see Fig 2). PCA was calculated examining 57 SNPs found associated to migratory behavior.

**Table A. Metrixes of migratory behavior in caribou ecotypes.**

| **Subspecies/**  **Ecotype** | **Seasonal ranges overlap**  **(mean; median; sd)** | **Departure timing of migration (mean; median; sd)** | **Distance of migration (mean; median; sd)** | **Latitudinal shift of migration**  **(mean; median; sd)** |
| --- | --- | --- | --- | --- |
| Barren-ground | 0.069; 0.05; 0.08 | May 6; May First; 66.411 | 247.46 km; 245.46 km; 90.83 | -1.43; -1.58; 1.01 |
| Woodland/  Boreal | 0.29; 0.24; 0.18 | April 11; March 28; 45.59 | 10.31 km; 9.02 km; 6.47 | 0.001; -0.003; 0.07 |
| Woodland/  Northern Mountain | 0.10; 0; 0.15 | April 24; April 15; 59.21 | 42.92 km; 31.56 km, 41.01 | -0.09; -0.04 0.23 |
|  |  |  |  |  |

Mean, median and standard deviation of individual caribou’s seasonal ranges overlap, distance and departure timing of migration for caribou subspecies and ecotypes distributed across western North America. Radio-collared caribou belonged to Barren-ground, Boreal and Northern Mountain ecotypes.

**Table B. SNPs associated to migratory behavior in caribou.**

|  | **Chr** | | **Pos** | **Ref/Alt** | **Loc.** | **Gene** | **β** | **Std. Error** | ***P*val** | **MAF** | **Relative PVE** |
| --- | --- | --- | --- | --- | --- | --- | --- | --- | --- | --- | --- |
| **IO** | *5* | *70142958* | | *T/C* | *intron* | *POLR3B* | *2.89E-01* | *5.22E-02* | *1.99E-07* | 0.080 | 20.9% |
|  | *9* | *23175623* | | *G/C* | *intron* | *UBE3D* | *3.17E-01* | *4.07E-02* | *3.35E-12* | 0.096 | 34.4% |
|  | *12* | *68665669* | | *C/G* | *intron* | *GPC6* | *3.17E-01* | *7.11E-02* | *7.06E-07* | 0.052 | 14.6% |
| **NSD classification** | *1* | *15434733* | | *T/C* | *inter* | *NA* | *-4.91E-01* | *9.56E-02* | *1.53E-06* | 0.129 | 1.6% |
|  | *1* | *83433341* | | *A/G* | *intron* | *THPO* | *-8.94E-01* | *1.75E-01* | *1.80E-06* | 0.069 | 1.6% |
|  | *2* | *47383588* | | *G/A* | *intron* | *KIF5C* | *-4.86E-01* | *7.49E-02* | *3.90E-09* | 0.190 | 2.3% |
|  | *2* | *62928091* | | *T/C* | *intron* | *TMEM163* | *-9.72E-01* | *1.90E-01* | *1.69E-06* | 0.054 | 1.6% |
|  | *2* | *67611642* | | *C/T* | *inter* | *NA* | *-5.28E-01* | *1.02E-01* | *1.17E-06* | 0.054 | 1.7% |
|  | *2* | *73376786* | | *G/C* | *inter* | *NA* | *-7.38E-01* | *1.33E-01* | *2.43E-07* | 0.089 | 1.8% |
|  | *2* | *130707598* | | *A/G* | *inter* | *NA* | *-5.11E-01* | *9.46E-02* | *4.82E-07* | 0.216 | 1.8% |
|  | *2* | *134928965* | | *G/A* | *inter* | *NA* | *-9.32E-01* | *1.69E-01* | *3.19E-07* | 0.052 | 1.8% |
|  | *4* | *5410284* | | *A/G* | *inter* | *NA* | *-6.12E-01* | *1.11E-01* | *3.10E-07* | 0.157 | 1.8% |
|  | *4* | *90192336* | | *C/A* | *inter* | *NA* | *-9.58E-01* | *1.87E-01* | *1.63E-06* | 0.053 | 1.6% |
|  | *5* | *26584231* | | *A/G* | *intron* | *ATF7* | *-6.25E-01* | *1.20E-01* | *1.20E-06* | 0.072 | 1.6% |
|  | *5* | *43321868* | | *C/T* | *intron* | *KCNMB4* | *-6.61E-01* | *1.28E-01* | *1.27E-06* | 0.099 | 1.6% |
|  | *5* | *106920863* | | *C/G* | *inter* | *NA* | *-9.44E-01* | *1.47E-01* | *5.84E-09* | 0.080 | 2.3% |
|  | *6* | *35059576* | | *C/T* | *inter* | *NA* | *-6.06E-01* | *1.12E-01* | *4.62E-07* | 0.105 | 1.8% |
|  | *6* | *47243160* | | *G/A* | *inter* | *NA* | *-7.29E-01* | *1.10E-01* | *2.36E-09* | 0.059 | 2.4% |
|  | *7* | *37716186* | | *A/T* | *inter* | *NA* | *-5.98E-01* | *9.92E-02* | *3.25E-08* | 0.122 | 2.1% |
|  | *7* | *47075725* | | *C/A* | *intron* | *TCF7* | *-8.88E-01* | *1.66E-01* | *6.00E-07* | 0.053 | 1.7% |
|  | *7* | *47361289* | | *A/C* | *inter* | *NA* | *-9.35E-01* | *1.69E-01* | *2.88E-07* | 0.079 | 1.8% |
|  | *9* | *60904848* | | *T/G* | *inter* | *NA* | *-5.16E-01* | *8.81E-02* | *6.71E-08* | 0.225 | 2.0% |
|  | *9* | *90760255* | | *T/C* | *inter* | *NA* | *-7.56E-01* | *1.42E-01* | *6.79E-07* | 0.111 | 1.7% |
|  | *9* | *92449044* | | *G/A* | *inter* | *NA* | *-7.03E-01* | *1.16E-01* | *2.96E-08* | 0.057 | 2.1% |
|  | *10* | *86801611* | | *A/G* | *intron* | *TMED10* | *-3.98E-01* | *7.18E-02* | *2.68E-07* | 0.337 | 1.8% |
|  | *11* | *1577006* | | *C/T* | *inter* | *NA* | *-8.85E-01* | *1.60E-01* | *2.95E-07* | 0.073 | 1.8% |
|  | *11* | *49610274* | | *C/T* | *exon* | *BUB1* | *-9.58E-01* | *1.87E-01* | *1.63E-06* | 0.062 | 1.6% |
|  | *11* | *100729657* | | *G/A* | *exon* | *HMCN2* | *-9.23E-01* | *1.50E-01* | *1.85E-08* | 0.061 | 2.1% |
|  | *12* | *21364754* | | *C/T* | *inter* | *NA* | *-9.41E-01* | *1.48E-01* | *6.66E-09* | 0.074 | 2.2% |
|  | *12* | *87132525* | | *C/T* | *intron* | *FAM155A* | *-7.67E-01* | *1.41E-01* | *3.93E-07* | 0.052 | 1.8% |
|  | *13* | *55241627* | | *G/A* | *inter* | *NA* | *-7.69E-01* | *1.44E-01* | *6.40E-07* | 0.087 | 1.7% |
|  | *14* | *34726617* | | *G/T* | *intron* | *C14H8orf34* | *-6.85E-01* | *1.25E-01* | *3.64E-07* | 0.183 | 1.8% |
|  | *14* | *68512840* | | *T/C* | *intron* | *MATN2* | *-5.73E-01* | *1.05E-01* | *3.73E-07* | 0.111 | 1.8% |
|  | *15* | *39944159* | | *G/C* | *intron* | *ARNTL* | *-7.19E-01* | *1.23E-01* | *6.60E-08* | 0.074 | 2.0% |
|  | *15* | *47259892* | | *A/G* | *exon* | *APBB1* | *-6.23E-01* | *9.03E-02* | *5.87E-10* | 0.053 | 2.5% |
|  | *15* | *68035100* | | *T/C* | *inter* | *NA* | *-8.52E-01* | *1.65E-01* | *1.41E-06* | 0.136 | 1.6% |
|  | *16* | *993508* | | *C/T* | *inter* | *NA* | *-8.73E-01* | *1.70E-01* | *1.44E-06* | 0.060 | 1.6% |
|  | *16* | *4334379* | | *A/G* | *intron* | *MAPKAPK2* | *-9.04E-01* | *1.62E-01* | *2.47E-07* | 0.090 | 1.8% |
|  | *16* | *30156981* | | *T/C* | *intron* | *PARP1* | *-8.72E-01* | *1.40E-01* | *1.41E-08* | 0.074 | 2.2% |
|  | *16* | *33890124* | | *G/A* | *inter* | *NA* | *-8.25E-01* | *1.49E-01* | *2.67E-07* | 0.058 | 1.8% |
|  | *16* | *48689198* | | *A/G* | *inter* | *NA* | *-8.62E-01* | *1.62E-01* | *7.27E-07* | 0.091 | 1.7% |
|  | *16* | *75398065* | | *A/G* | *inter* | *NA* | *-8.83E-01* | *1.73E-01* | *1.81E-06* | 0.063 | 1.6% |
|  | *17* | *70382111* | | *C/T* | *inter* | *NA* | *-5.47E-01* | *9.52E-02* | *1.14E-07* | 0.084 | 1.9% |
|  | *20* | *33469905* | | *A/G* | *exon* | *MROH2B* | *-6.66E-01* | *1.22E-01* | *3.98E-07* | 0.177 | 1.8% |
|  | *20* | *70809953* | | *G/T* | *inter* | *NA* | *-6.53E-01* | *1.28E-01* | *1.62E-06* | 0.089 | 1.6% |
|  | *21* | *61369229* | | *T/C* | *inter* | *NA* | *-7.60E-01* | *1.37E-01* | *2.44E-07* | 0.065 | 1.8% |
|  | *21* | *71025379* | | *A/G* | *inter* | *NA* | *-5.49E-01* | *9.42E-02* | *7.87E-08* | 0.085 | 2.0% |
|  | *22* | *52465777* | | *T/C* | *exon* | *DHX30* | *-9.77E-01* | *1.82E-01* | *5.61E-07* | 0.062 | 1.7% |
|  | *22* | *57528644* | | *C/A* | *inter* | *NA* | *-6.98E-01* | *1.35E-01* | *1.23E-06* | 0.083 | 1.6% |
|  | *24* | *37590712* | | *C/T* | *prom* | *LPIN2* | *-6.80E-01* | *1.24E-01* | *3.39E-07* | 0.093 | 1.8% |
|  | *26* | *30690738* | | *C/T* | *intron* | *XPNPEP1* | *-7.31E-01* | *1.38E-01* | *8.23E-07* | 0.057 | 1.7% |
|  | *26* | *42673045* | | *C/T* | *intron* | *HTRA1* | *-8.71E-01* | *1.58E-01* | *2.95E-07* | 0.065 | 1.8% |
|  | *26* | *51419599* | | *A/G* | *inter* | *NA* | *-4.84E-01* | *9.08E-02* | *6.74E-07* | 0.205 | 1.7% |
|  | *27* | *38508910* | | *T/C* | *intron* | *PSD3* | *-9.23E-01* | *1.26E-01* | *8.88E-11* | 0.067 | 2.7% |
|  | *29* | *11548297* | | *C/T* | *inter* | *NA* | *-5.51E-01* | *9.92E-02* | *2.62E-07* | 0.050 | 1.8% |
|  | *29* | *47946501* | | *G/A* | *intron* | *ANO1* | *-7.50E-01* | *1.38E-01* | *4.12E-07* | 0.053 | 1.8% |
|  | *X* | *64642821* | | *T/C* | *intron* | *PAK3* | *-6.19E-01* | *1.15E-01* | *5.27E-07* | 0.100 | 1.7% |

Information for SNPs found significantly associated with migratory behavior (Index of overlap - IO – or Net Square Displacement –NSD), including: chromosome position; location (intergenic, intron, exon or within a promoter); annotation (name of gene harboring the SNP); Beta coefficients (*β*) of association, standard error and *P-value*; minor allele frequency (MAF); and relative proportion of phenotypic variance (*PVE*).

**Table C. Differentiation of minor allele frequencies (*MAF*) between caribou genetic clusters (K) detected with the *Admixture* program.**

|  | North (K2) | South (K2) | North (K3) | Mountain (K3) | Boreal (K3 |
| --- | --- | --- | --- | --- | --- |
| North (K2)  (mean *MAF*=0.07) | - | - |  |  |  |
| South(K2)  (mean *MAF*=0.12) | **chi-squared = 23.18; *p*=1.5x10^-6^** | - |  |  |  |
| North(K3)  (mean *MAF*=0.07) |  |  | - | - | - |
| Mountain (K3)  (mean *MAF*=0.16) |  |  | **chi-squared = 40.04; *p*=2.5x10^-10^** | - | - |
| Boreal (K3)  (mean *MAF*=0.09) |  |  | chi-squared = 0.66; *p*=0.42 | **chi-squared = 26.49; *p*=2.6x10^-7^** | - |
|  |  |  |  |  |  |

Significant results are highlighted in bold (Kruskal-Wallis test). Pairwise comparisons are reported only between clusters obtained with the same K (2 or 3).

**Table D. Dependence of individual caribou classification as migrant upon ancestry, while statistically controlling for ecotype.**

| **Model (Logit (NSD) ~** | **β** | **Std. Error** | **z value** | **Pr(>\|z\|)** | **AIC** |
| --- | --- | --- | --- | --- | --- |
| **Admixture proportion (Q) *–* K=2** | | | | | |
| **North** | **1.808** | **0.838** | **2.157** | **0.031** | **79.569** |
| *Condition dependence upon ecotype belonging* | | | | | |
| North+ (1\|ecotype) | 1.714 | 0.956 | 1.794 | 0.073 | 81.268 |
| **Admixture proportion (Q) *–* K=3** | | | | | |
| Boreal | -0.976 | 0.831 | -1.174 | 0.240 | 82.794 |
| **North** | **1.768** | **0.767** | **2.306** | **0.021** | **78.841** |
| **Mountain** | **-2.142** | **1.044** | **-2.051** | **0.040** | **80.299** |
| *Condition dependence upon ecotype belonging* | | | | | |
| Boreal + (1\|ecotype) | -0.787 | 1.042 | -0.755 | 0.450 | 83.462 |
| **North + (1\|ecotype)** | **1.686** | **0.853** | **1.975** | **0.048** | **80.693** |
| Mountain + (1\|ecotype) | -1.765 | 1.173 | -1.505 | 0.132 | 81.834 |
|  | | | | | |

Results of logistic regression analyses examining dependence of Net Square Displacement classification of migrants upon admixture proportion (*Q*) to two or three caribou clusters (K); Beta coefficient (β), Standard error, *z* value, *p*-value and AIC are indicated, while the models in parenthesis account for caribou ecotype as a random effect. Models in bold are significant; NSD= classification of animals as migratory (1) vs. sedentary (0).

## References

1. Ali OA, O’Rourke SM, Amish SJ, Meek MH, Luikart G, Jeffres C, et al. RAD capture (Rapture): flexible and efficient sequence-based genotyping. Genetics. 2016;202: 389-400. Available from: https://doi.org/10.1534/genetics.115.183665
2. Catchen J, Hohenlohe PA, Bassham S, Amores A, Cresko WA. Stacks: an analysis tool set for population genomics. Mol Ecol. 2013;22: 3124-3140. Available from: https://doi.org/10.1111/mec.12354
